# How Health Care Workers Wield Influence Through Twitter Hashtags: Retrospective Cross-sectional Study of the Gun Violence and COVID-19 Public Health Crises

**DOI:** 10.2196/24562

**Published:** 2021-01-06

**Authors:** Ayotomiwa Ojo, Sharath Chandra Guntuku, Margaret Zheng, Rinad S Beidas, Megan L Ranney

**Affiliations:** 1 Harvard Medical School Boston, MA United States; 2 Penn Medicine Center for Digital Health Philadelphia, PA United States; 3 Department of Computer and Information Science University of Pennsylvania Philadelphia, PA United States; 4 Leonard Davis Institute of Health Economics University of Pennsylvania Philadelphia, PA United States; 5 Department of Psychiatry University of Pennsylvania Philadelphia, PA United States; 6 Brown-Lifespan Center for Digital Health Brown University Providence, RI United States

**Keywords:** COVID-19, firearm injury, social media, online advocacy, Twitter, infodemiology, infoveillance, tweet, campaign, health care worker, influence, public health, crisis, policy

## Abstract

**Background:**

Twitter has emerged as a novel way for physicians to share ideas and advocate for policy change. #ThisIsOurLane (firearm injury) and #GetUsPPE (COVID-19) are examples of nationwide health care–led Twitter campaigns that went viral. Health care–initiated Twitter hashtags regarding major public health topics have gained national attention, but their content has not been systematically examined.

**Objective:**

We hypothesized that Twitter discourse on two epidemics (firearm injury and COVID-19) would differ between tweets with health care–initiated hashtags (#ThisIsOurLane and #GetUsPPE) versus those with non–health care–initiated hashtags (#GunViolence and #COVID19).

**Methods:**

Using natural language processing, we compared content, affect, and authorship of a random 1% of tweets using #ThisIsOurLane (Nov 2018-Oct 2019) and #GetUsPPE (March-May 2020), compared to #GunViolence and #COVID19 tweets, respectively. We extracted the relative frequency of single words and phrases and created two sets of features: (1) an open-vocabulary feature set to create 50 data-driven–determined word clusters to evaluate the content of tweets; and (2) a closed-vocabulary feature for psycholinguistic categorization among case and comparator tweets. In accordance with conventional linguistic analysis, we used a *P*<.001, after adjusting for multiple comparisons using the Bonferroni correction, to identify potentially meaningful correlations between language features and outcomes.

**Results:**

In total, 67% (n=4828) of #ThisIsOurLane tweets and 36.6% (n=7907) of #GetUsPPE tweets were authored by health care professionals, compared to 16% (n=1152) of #GunViolence and 9.8% (n=2117) of #COVID19 tweets. Tweets using #ThisIsOurLane and #GetUsPPE were more likely to contain health care–specific language; more language denoting positive emotions, affiliation, and group identity; and more action-oriented content compared to tweets with #GunViolence or #COVID19, respectively.

**Conclusions:**

Tweets with health care–led hashtags expressed more positivity and more action-oriented language than the comparison hashtags. As social media is increasingly used for news discourse, public education, and grassroots organizing, the public health community can take advantage of social media’s broad reach to amplify truthful, actionable messages around public health issues.

## Introduction

Twitter has emerged as a novel way for physicians to organize and advocate for policy change, and combat misinformation amid national health crises. One in 5 adults in the United States uses Twitter, and 75% report using this platform as a news outlet [1]. When Twitter advocacy campaigns brand their movement with a hashtag, tagged tweets are easily archived and found, opening up discussions to users who do not have any personal connection to the authors.

#ThisIsOurLane and #GetUsPPE are examples of health care–initiated Twitter movements that went viral. In November 2018, in response to the National Rifle Association’s tweet asserting that “Someone should tell self-important anti-gun doctors to stay in their lane…,” Dr Michael Gonzalez coined #ThisIsOurLane to describe why health care professionals are involved in firearm injury prevention and treatment [2]. During the COVID-19 pandemic, Dr Esther Choo initiated #GetMePPE, later expanded to #GetUsPPE, to raise awareness about critical personal protective equipment (PPE) shortages [3]. Anecdotes suggest #ThisIsOurLane influenced societal perceptions of health care professionals’ role in firearm injury [4], and #GetUsPPE galvanized attention to hospitals’ unmet PPE needs [5,6].

Whether online discussions with health care–initiated hashtags actually differ from contemporaneous discussions of the firearm injury and COVID-19 epidemics has not been quantified. Nor, to our knowledge, has the involvement of Twitter users outside of health care been examined. Understanding the content and voice of health professionals on social media during public health crises is essential. Rampant misinformation about health care online has led to international debates about how best to change public knowledge and conversations. At the same time, some experts are bemoaning “infodemics,” in which people are so overwhelmed by contradictory facts that they become unable to act to protect themselves and their families [7]. Examining the content, tone, and types of tweeters involved in health care–led social media campaigns could inform future efforts related to data dissemination by the medical and nonmedical community [8].

To examine the characteristics of these online discussions, we compared psycholinguistic characteristics (ie, content and affect) of tweets among two cohorts: contemporaneous tweets regarding gun violence (comparing tweets with #ThisIsOurLane vs #GunViolence) and contemporaneous tweets regarding the COVID-19 pandemic (#GetUsPPE/#GetMePPE vs #COVID19). We hypothesized that messages using health care–led hashtags would be more negative in tone (reflecting frustration and negative directives) but also more actionable in content (providing solutions) compared with non–health care–related hashtags, given health care professionals’ personal stake and proximity to these issues.

## Methods

This retrospective cross-sectional study selected a random 1% sample of publicly available Twitter data containing specific hashtags from across the United States.

### Data

**Figure 1:**
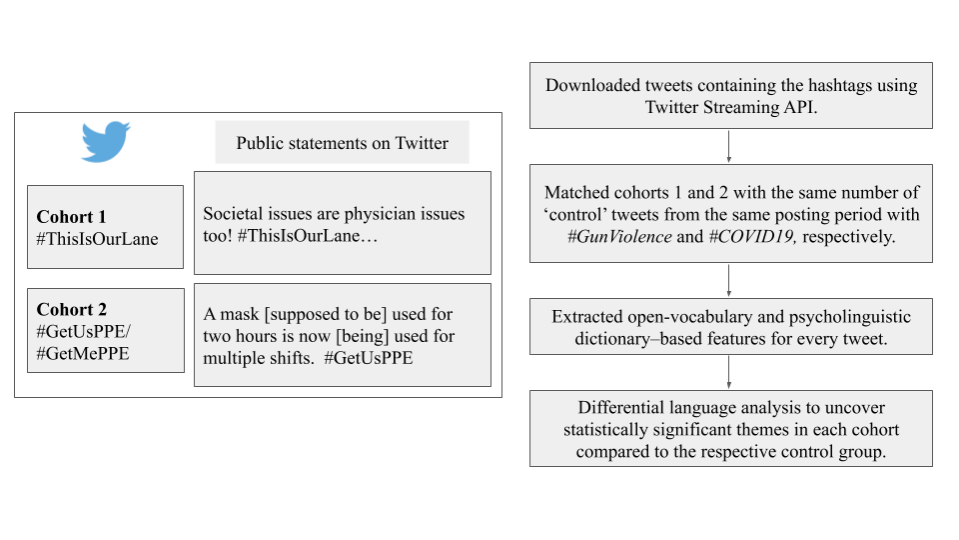
Study flowchart. API: application programming interface.

For cohort 1, we identified tweets containing #ThisIsOurLane (n=38,774) or #GunViolence (n=52,183) between November 7, 2018, and October 13, 2019, given multiple episodes of gun violence with national attention during this time period. For cohort 2, we identified tweets with #GetUsPPE or #GetMePPE (n=39,658) or #COVID19 (n=200,000) between March 17, 2020, and May 20, 2020, which reflects the duration of the campaign at the time of the analysis. Both study periods began when the hashtag was introduced. After discarding retweets and tweets containing only hashtags and user mentions (without any other words), 7201 #ThisIsOurLane tweets and 21,605 #GetUsPPE/#GetMePPE tweets remained as "cases". Tweets containing both case and control hashtags were preserved as cases in the analysis. A random sample of 7201 of the remaining #GunViolence-only tweets and 21,605 of the #COVID19-only tweets were selected as comparators for two separate analyses (Figure 1). Although tweets about gun violence and COVID-19 used other hashtags, these were identified as trending and potentially the most common around the study period and were used as comparators.

We used the Python package TwitterMySQL [9], which utilizes the official Twitter application programming interface (API), to collect tweets matching at least one of the keywords described above in real time. We note that the Twitter API limits such streams to 1% of the total Twitter volume at any given moment. Similar methods have been used in prior work studying health-related tweets [10-14].

We obtained Twitter profile descriptions of the users in our data set using the Twitter API and searched for words indicating health care professional status using regular expressions (eg, “doc*,” “medic*,” “surg*”). When processing tweets for this analysis, we only utilized information publicly available on users’ Twitter profiles. The University of Pennsylvania Institutional Review Board deemed the study exempt.

### Extracting Language Features

After tokenizing the tweets [15], we extracted the relative frequency of single words and phrases and created two sets of features: (1) an open vocabulary feature set [16] defined using the MALLET (Machine Learning for Language Toolkit) implementation of latent Dirichlet allocation [17], an unsupervised clustering algorithm, to create 50 data-driven–determined word clusters; and (2) a closed vocabulary feature set defined as the normalized frequency of 71 psycholinguistic categories among case and comparator tweets, created with Linguistic Inquiry Word Count dictionary [18].

### Statistical Analysis

Each feature set was input in a logistic regression model, with “case” (ie, #ThisIsOurLane or #GetUsPPE) as the dependent variable. In accordance with conventional linguistic analysis, we used a *P* value of <.001, after adjusting for multiple comparisons using the Bonferroni correction, to identify potentially meaningful correlations between language features and outcomes. We calculated regression coefficients with the #GunViolence and #COVID19 (comparator) groups as references.

## Results

In total, 67% (n=4828) of #ThisIsMyLane tweets and 36.6% (n=7907) of #GetUsPPE tweets were authored by health care professionals, compared to 16% (n=1152) of #GunViolence and 9.8% (n=2117) of #COVID19 tweets.

**Figure 2:**
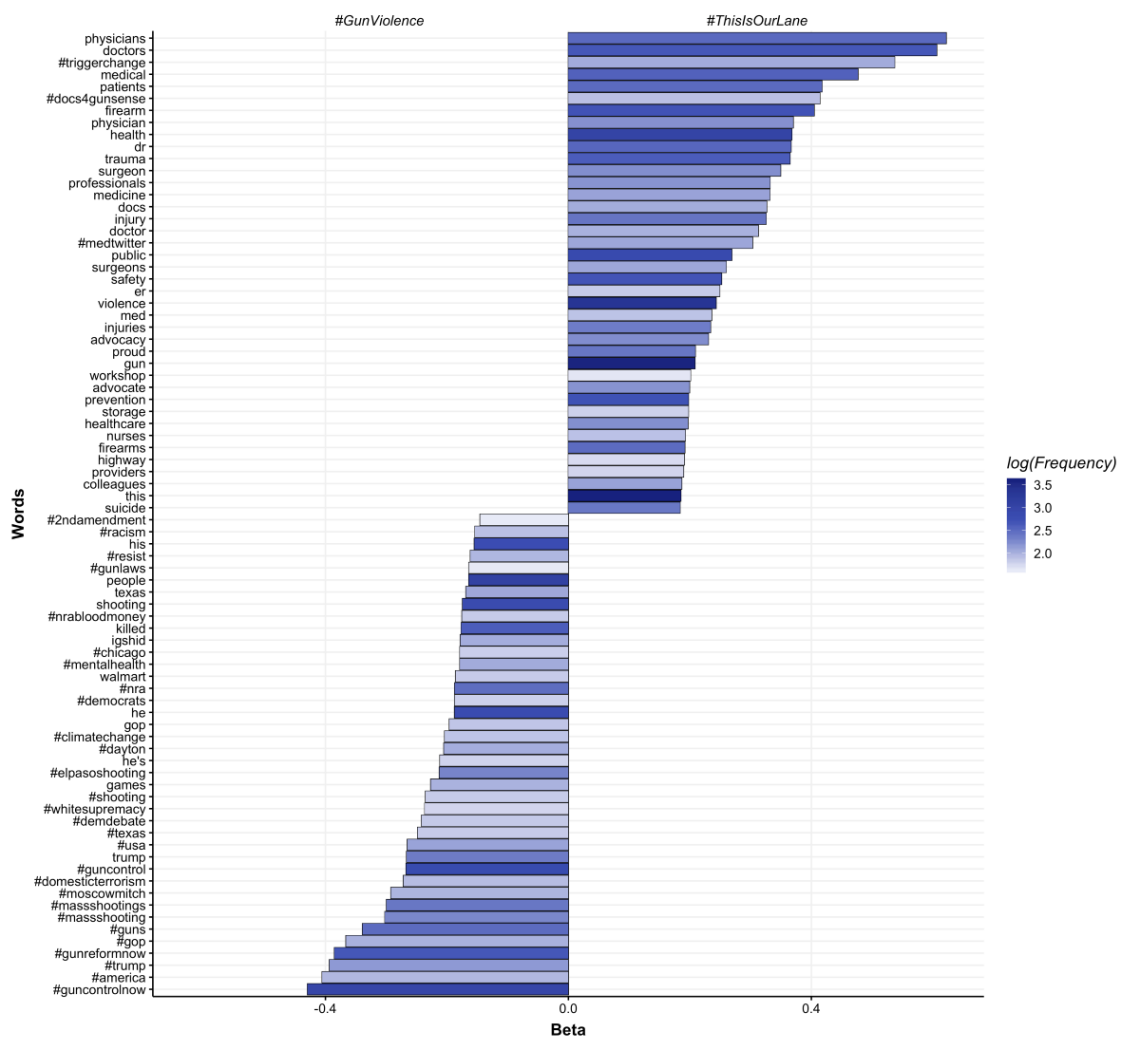
Words associated with #ThisIsOurLane tweets compared to #GunViolence. Beta indicates the strength of association of each word with respective groups and color indicates frequency. All words are statistically significant at p<.05, Benjamin Hochberg correction.

**Figure 3:**
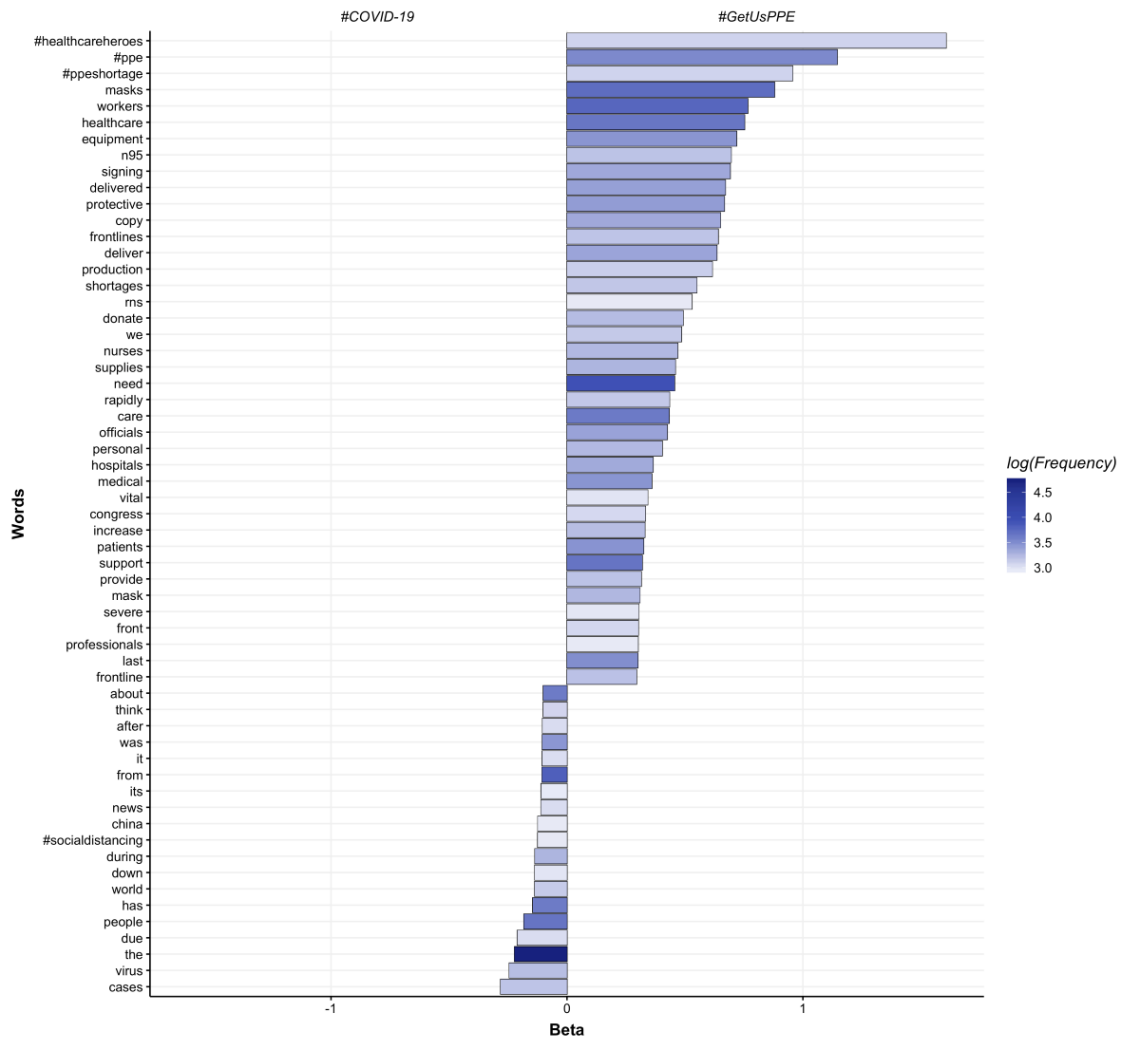
Words associated with #GetUsPPE tweets compared to #COVID19. Beta indicates the strength of association of each word with respective groups and color indicates frequency. All words are statistically significant at p<.05, Benjamin Hochberg correction.

**Figure 4:**
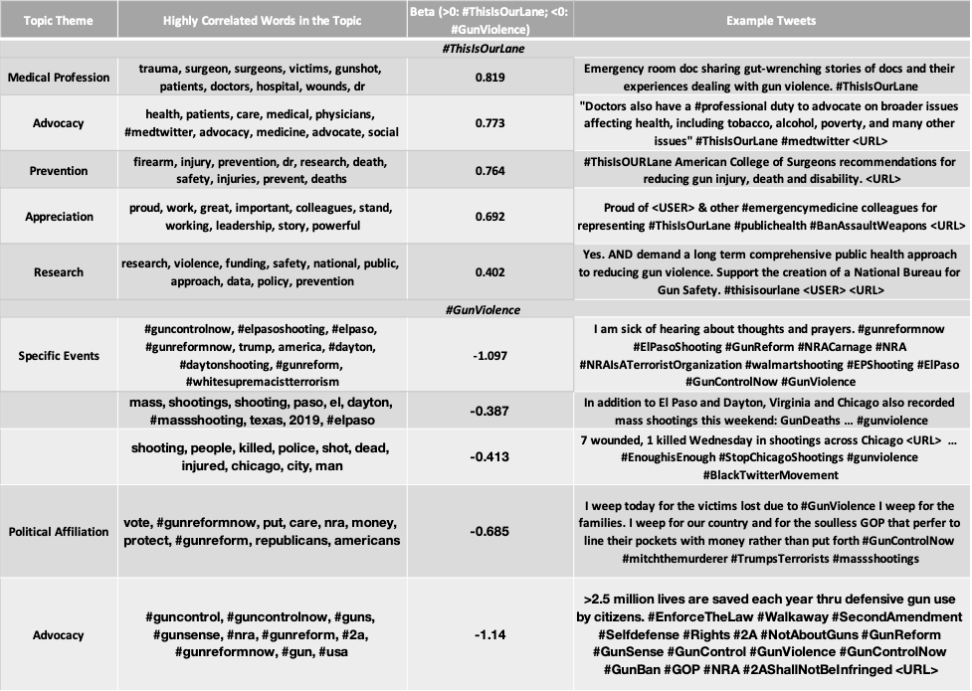
Highly correlated topics with mention of #ThisIsOurLane vs. #GunViolence. Beta indicates the strength of association of each topic. Top words and example paraphrased tweets for each topic are shown. Topics are statistically significant at p<.05, Benjamin Hochberg correction.

**Figure 5:**
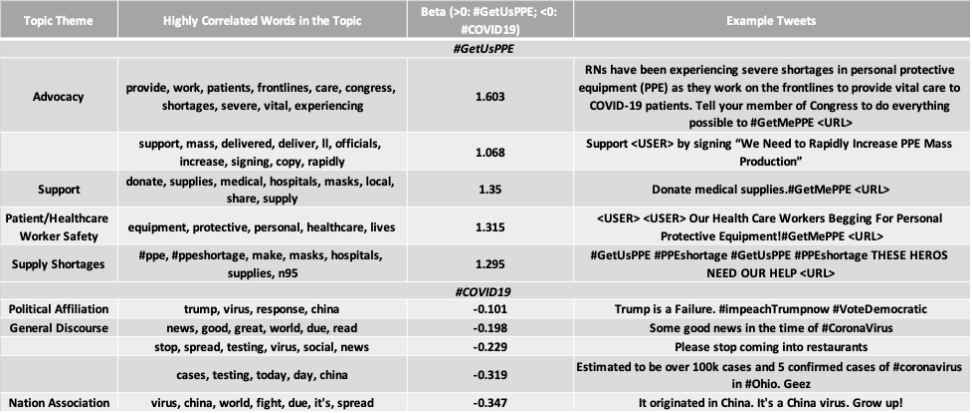
Highly correlated topics with mention of #GetUsPPE vs. #COVID19. Beta indicates the strength of association of each topic. Top words and example paraphrased tweets for each topic are shown. Topics are statistically significant at p<.05, Benjamin Hochberg correction.

The open-vocabulary feature set (ie, content) of #ThisIsOurLane and #GetUsPPE were more likely to contain language specific to health care than general tweets using hashtags #GunViolence and #COVID19 (Figures 2-5). Specifically, #ThisIsOurLane tweets discussed health care professionals’ advocacy, research, or appreciation of colleagues, and were more likely to mention public health and community compared with #GunViolence tweets. #ThisIsOurLane tweets were less likely to mention political entities like #NRA and specific events such as #ElPaso. #GetUsPPE tweets described severe PPE shortages for health care workers, the need to support patient and staff safety, and referenced health care workers as heroes. Additionally, #GetUsPPE tweets included more action-oriented language (ie, deliver, sign, support) compared with #COVID19 tweets.

**Figure 6:**
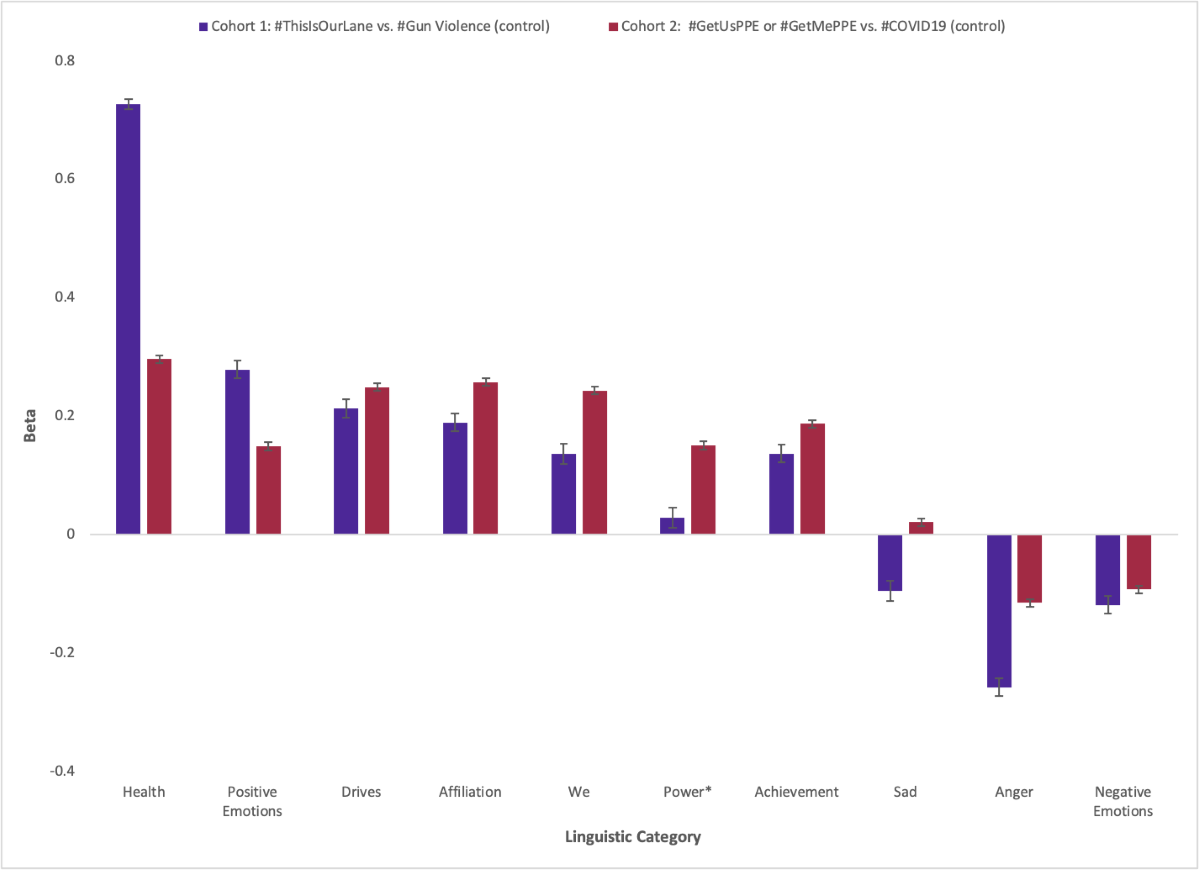
Linguistic correlates of health care–led Twitter hashtag campaigns (#ThisIsOurLane and #GetUsPPE/#GetMePPE) compared with general ones (#GunViolence and #COVID19). Positive beta indicates a strong correlation of the linguistic category with the case compared to the control tweets. *“Power” was not significant at *P*<.001 for cohort 1.

Analysis of closed-vocabulary associations (ie, psycholinguistic categories) demonstrated that tweets with #ThisIsOurLane or #GetUsPPE contained more language associated with health, positive emotions, affiliation, and group identity compared to tweets with #GunViolence or #COVID19, respectively (Figure 6). General tweets about gun violence and the COVID-19 pandemic contained more words associated with negative emotions or anger than tweets with health care–initiated hashtags.

## Discussion

### Principal Findings

This study demonstrates not just the reach but also the inclusiveness and uniqueness of tweets containing health care–led hashtags about commonly discussed health care epidemics. Consistent with our hypotheses, tweets containing health care–led hashtags differed qualitatively and quantitatively from other tweets on the same topic during the same time period, albeit not in the way that we predicted. Tweets with #ThisIsOurLane and #GetUsPPE expressed more positivity and a greater sense of group affiliation than comparison hashtags led by the general public. Both #ThisIsOurLane and #GetUsPPE tweets contained more actionable language such as “research,” “prevention,” and “support.“

Social media’s potential as a platform for enhancing health discussions is frequently discussed [19,20]. Some authors have even urged the use of social media to develop grassroots “new power” movements that can combat mistruths in science and public health [21]. Others have described the potential utility of specific health care–led tweets for disseminating factual information [22]. Our analysis supports that health care–led hashtags contribute unique, actionable content and tone to national discussions about health, and can create new, inclusive movements that provide opportunities for health care professionals and non–health care–based individuals. Although we did not examine the relative prevalence of facts versus misinformation between the two sets of hashtags, the results of our study offer further evidence of the value of using Twitter to shape and build support for public health movements.

Prior literature demonstrates social media’s potential for reaching new groups regarding issues in medicine and public health. However, few previous studies have characterized whether the content of social media campaigns initiated by the health care community are truly unique. For example, TikTok videos about COVID-19 accumulated over 1 billion views; however, an analysis of these videos reports that only a small portion were led by health care professionals, and that few—even those developed by the World Health Organization—included actionable tools to prevent or handle the pandemic [23,24]. Another study reported that a Twitter campaign to raise skin cancer prevention awareness led to nearly 12 million impressions on social media [25] but did not examine content or tone of shared posts. Still, others have demonstrated that health-related content on social media reflects local public health concerns and sentiments but have not examined the relative contribution of health care– versus non–health care–led hashtags [26-28]. Our work is therefore unique in examining not only the number of posts but also what differentiated them from non–health care–led posts on the same topics at the same time.

A particularly noteworthy finding from our study is the positive tone and action-oriented content of tweets with health care–initiated hashtags. This finding differs from our expectations: we hypothesized that health care professionals would be sharing the truth about firearm injury and COVID-19, and that these realities would be negatively valenced. The finding of positive tone, even on difficult issues, may reflect societal expectations of professionalism from medical experts [29,30]. It may also reflect health care professionals’ desire to motivate action in others: positive affect and positive tone both increase the acceptability and efficacy of behavioral interventions [31,32]. Indeed, some work has specifically provided guidance to health care and public health professionals on how to avoid or manage “trolls” [33]. Future work should examine whether successful hashtag campaigns are more positive than unsuccessful campaigns.

Establishing hashtags makes health care professionals’ conversations more accessible to the nonmedical community and can be used to cultivate momentum around public health campaigns that carry educational and actionable content. Despite #ThisIsOurLane and #GetUsPPE being initiated and more commonly used by health care professionals, people outside of health care also commonly tweeted with these terms. Based on hashtag categories developed by Saxton et al [34], #ThisIsOurLane and #GetUsPPE are public education and call-to-action hashtags, which are most likely to be retweeted, and therefore most effective for online advocacy.

Future work should examine the characteristics of successful hashtag development and dissemination, as how to best create and shepherd these discussions is undetermined. Based on the origin story of #GetUsPPE and #ThisIsOurLane, a successful movement likely does not depend on derivation from a large company or influential organization. Instead, as Twitter increasingly serves as a news source for the general public [35], it offers a platform for average health care professionals to both spread facts and increase action on critical public health issues. Some works in the literature have developed best practices for successfully using health care hashtags to increase audience engagement [34]. Although the United States’ Centers for Disease Control and Prevention has guidelines on Twitter use for health communication, initial analyses suggest mixed efficacy of their Twitter campaigns [36]. To inform others’ work, future research should examine in more detail which characteristics of #ThisIsOurLane and #GetUsPPE enabled coalescence of a larger community.

### Limitations

Limitations to this analysis include the correlational and noncausal nature of the results. This study cannot comment on whether health care–led hashtag campaigns introduced new thoughts on national health issues, as we did not review tweets from health care professionals about gun violence or the COVID-19 pandemic before the hashtags were introduced. Additionally, the magnitude of the influence of tweets with health care–led hashtags is not characterized. Finally, our analysis did not account for the voice of patients and survivors, who have previously been shown to have a powerful role on Twitter.

### Conclusion

Historically, health care professionals play defining roles in social justice and public health movements. Health care–led hashtag campaigns are positive, actionable, and portray a united front in developing solutions to pressing public health issues. The #ThisIsOurLane and #GetUsPPE movements exemplify how online media can influence 21st-century social dialogues about disease, injury, and prevention.

## Abbreviations

API: application programming interface
MALLET: Machine Learning for Language Toolkit
PPE: personal protective equipment
